# Comparison of Esophageal Function Tests in Chinese Patients with Functional Heartburn and Reflux Hypersensitivity

**DOI:** 10.1155/2017/3596148

**Published:** 2017-09-06

**Authors:** Feng Gao, Yan Gao, Xue Chen, Jie Qian, Jie Zhang

**Affiliations:** ^1^Digestive Department, Beijing Anzhen Hospital, Capital Medical University, Beijing 100029, China; ^2^Digestive Department, Beijing Chao-Yang Hospital, Capital Medical University, Beijing 100020, China

## Abstract

**Objective:**

To investigate the differences in the results of esophageal function tests for functional heartburn (FH) and reflux hypersensitivity (RH).

**Methods:**

Patients with FH and RH and healthy volunteers (HVs) from the Department of Gastroenterology, Beijing Anzhen Hospital and Beijing Chao-Yang hospital, who underwent high-resolution manometry and impedance (HRIM), and 24-hour multichannel intraluminal impedance and pH recording (MII/pH) between 2014 and 2016, were enrolled in this study.

**Results:**

36 HV, 147 FH patients, and 91 RH patients were enrolled. The postreflux swallow-induced peristaltic wave index (PSPW index) and mean nocturnal baseline impedance (MNBI) values were significantly lower in RH than in FH and HV. The ineffective esophageal motility (IEM), fragmented peristalsis rates, total bolus exposure, proximal total reflux events, and distal total reflux events were significantly greater in RH than in FH and HV.

**Conclusions:**

Compared to HV and FH patients, RH patients exhibited greater IEM and fragmented peristalsis rates, a greater total bolus exposure, more proximal total and distal total reflux events, and reduced chemical clearance and mucosal integrity. By using the above described parameters, HRIM and MII/pH assays could be used to correctly classify RH and FH and hence allow physicians to provide adequate relief from associated symptoms.

## 1. Introduction

### 1.1. FH

Functional heartburn (FH) can be defined by reflux symptoms (such as retrosternal pain or acid reflux) in the absence of gastroesophageal reflux disease (GERD), histopathological mucosal abnormalities, major disorders, or structural explanations. Reflux hypersensitivity (RH) is assessed to identify patients with esophageal symptoms (heartburn or chest pain) that could be considered within the realm of GERD clinically, without evidence of reflux on endoscopy or pH-impedance monitoring, but with demonstrable triggering of symptoms with physiological reflux [1].

With the clinical application of 24-hour multichannel intraluminal impedance and pH recording (MII/pH), patients with heartburn and normal upper gastrointestinal endoscopy can be classified into abnormal acid exposure, FH, and RH. Patients with heartburn without objective evidence of reflux (normal upper gastrointestinal endoscopy, normal esophageal acid exposure, and no correlation between symptoms and reflux events) are diagnosed with FH. By contrast, patients with heartburn, normal upper gastrointestinal endoscopy, and normal esophageal acid exposure, but a positive correlation between symptoms and reflux events (symptom index > 50% or a symptom association probability > 95%), are diagnosed with RH [1–5]. However, few studies have compared patients with FH and RH [4, 6, 7], and no study has explored the difference between FH and RH in Chinese patients. Therefore, we investigated the differences in the results of esophageal function tests between FH and RH in Chinese patients.

## 2. Methods

### 2.1. Ethics

The study was approved by the Ethics Board of Beijing Anzhen Hospital, Capital Medical University. All participants gave written informed consent.

### 2.2. Patient Selection

Chinese patients who presented with symptoms of heartburn with normal upper gastrointestinal endoscopy and who underwent high-resolution manometry and impedance (HRIM) and 24-hour multichannel intraluminal impedance and pH recording (MII/pH) at the Department of Gastroenterology of Beijing Anzhen Hospital or at Beijing Chao-Yang Hospital between December 2014 and December 2016 were enrolled. Healthy volunteers (HVs) enrolled during the same period were also included. Patients with other chronic active medical diseases (such as coronary artery disease, hypertension, malignancy, and diabetes mellitus) were excluded.

The diagnosis of FH was made based on the following criteria: (i) presence of heartburn as the predominant symptom, (ii) no evidence of gastroesophageal reflux (normal MII/pH monitoring results) or eosinophilic esophagitis as the cause of the symptoms, (iii) no major esophageal motor disorders (achalasia/esophagogastric junction (EGJ) outflow obstruction, diffuse esophageal spasm, jackhammer esophagus, or absent peristalsis), and (iv) presence of FH for the last 3 months, with symptom onset at least 6 months before diagnosis at a frequency of at least twice a week [1–3].

A diagnosis of RH required all of the following criteria: (i) retrosternal symptoms including heartburn and chest pain, (ii) normal endoscopy and no evidence that eosinophilic esophagitis is the cause of the symptoms, (iii) no major esophageal motor disorders (achalasia/EGJ outflow obstruction, diffuse esophageal spasm, jackhammer esophagus, or absent peristalsis), and (iv) evidence of reflux events triggering symptoms despite normal acid exposure according to pH or pH-impedance monitoring (a response to antisecretory therapy did not exclude a diagnosis). These criteria had to be met for the last 3 months, with symptom onset at least 6 months before diagnosis at a frequency of at least twice a week [1].

### 2.3. Stationary High-Resolution Esophageal Manometry and Impedance

A specially designed solid-state manometry catheter (Sandhill Scientific, Highland Ranch, CO, USA) with 32 manometric sensors and four pairs of MII sensors separated by 5 cm intervals was used to assess esophageal pressure and impedance with the patient in the supine position. The lower esophageal sphincter (LES) was examined using distal circumferential manometric sensors. The catheter was positioned such that the pressure transducers were located across the upper esophageal sphincter, esophageal body, and LES, and the distal channels were in the stomach. Ten swallows of 5 mL normal saline (0.9%) solution were then performed at 30-second intervals with the examinates in the supine position.

### 2.4. Twenty-Four-Hour Esophageal Multichannel Intraluminal Impedance and pH Recordings

The 2.1 mm outer diameter catheter comprised six electrode pairs measuring intraluminal impedance at 3, 5, 7, 9, 15, and 17 cm above the LES and an antimony pH sensor 5 cm above the LES (Sandhill Scientific). An impedance amplifier delivered an ultralow current at a range of 1-2 kHz, with resulting current flow variation in response to intraluminal impedance changes (high impedance indicates gas or air, and low impedance indicates liquid). The signals from six impedance channels and one pH channel were recorded at 50 samples per second. The data were stored using an ambulatory recorder and saved on a 256 MB CompactFlash card. Event markers were used to record the occurrence of symptoms, times of meals, and changes in body position. The study was performed in outpatients after an overnight fast, and the LES was located by esophageal manometry. The patients underwent HRIM and MII/pH after a 7-day washout of the proton pump inhibitors and H_2_ antagonists.

### 2.5. Data Collection

Esophageal bolus clearance can be assessed by measuring the total bolus transit time (TBTT) by classifying swallows as complete bolus transit (if bolus entry occurs at the most proximal site and bolus exit points are recorded in all three distal recording segments), or incomplete bolus transit (if bolus exit is not identified at any of the three distal recording segments), and the complete bolus transit rate (CBTR). The distal contractile integral (DCI) of the distal segmental contraction is a parameter that integrates contractile pressure (mmHg), the duration (s) of the contraction, and the length of the smooth muscle esophagus (cm). The distal esophageal amplitude (DEA) is an average of the contraction amplitudes at 5 and 10 cm above the LES. The integrated relaxation pressure (IRP) is the mean EGJ pressure measured using the electronic equivalent of a sleeve sensor for four continuous or noncontinuous seconds of relaxation in the 10-second window following deglutitive upper esophageal sphincter relaxation. The lower esophageal sphincter length (LESL), lower esophageal sphincter pressure (LESP), lower esophageal sphincter residual pressure (LESRP), and upper esophageal sphincter pressure (UESP) were also measured [8, 9]. Ineffective esophageal motility (IEM) is defined as at least 50% of swallows with a DCI < 450 mmHg/s/cm [10]. Fragmented peristalsis is defined as at least 50% fragmented swallows (contractions with DCI > 450 mmHg/s/cm and a break > 5 cm in the 20 mmHg isobaric contour) [10]. The acid exposure upright (%), acid exposure recumbent (%), acid exposure total (%), bolus exposure upright (%), bolus exposure recumbent (%), bolus exposure total (%), proximal acid events, proximal nonacid events, proximal total reflux events, distal acid reflux events, distal nonacid reflux events, and distal total reflux events were measured [11]. The DeMeester score was calculated. Symptoms were considered to be associated with reflux if they occurred within a 2-minute window after onset of the reflux event [11]. The symptom index was considered positive when ≥50%; the symptom association probability (SAP) was considered positive when ≥95% [12, 13]. All parameters were measured using Bio View Analysis software (Sandhill Scientific, Inc., Highland Ranch, CO, USA).

A postreflux swallow-induced peristaltic wave (PSPW) was defined as an antegrade 50% drop in impedance (relative to the preswallow baseline) originating in the most proximal impedance site, attaining all distal impedance sites, followed by a return to at least 50% of the baseline levels at the sites of distal impedance (bolus exit). Postreflux swallows that did not reach the distal impedance sites, or that occurred more than 30 s after the end of reflux episodes, were not considered. For each impedance-pH tracing event, the number of refluxes followed within 30 s by PSPWs was divided by the number of total refluxes (calculated manually) to obtain the PSPW index [14].

Mean nocturnal baseline impedance (MNBI) was assessed from the most distal impedance channel during nighttime recumbency. Three 10-minute time periods (at around 1:00 AM, 2:00 AM, and 3:00 AM) were selected, and the mean baseline for each period was computed with the aid of appropriate software. Time periods that included swallows, refluxes, and pH drops were avoided. The mean of the three measurements was manually calculated to obtain the MNBI [15].

### 2.6. Comparison Groups

We formed three study groups: Chinese HV, FH, and RH subjects.

### 2.7. Statistical Methods

Categorical data were described as numbers and continuous data as means ± SD. Data were compared between FH and RH patients using the independent sample *t*-test, chi-square test, or Fisher's exact test. Data were compared among the three groups by ANOVA or employing the chi-square or Fisher's exact test. Stepwise linear regression analyses were performed to explore the influence of all variables on RH and IEM. For each variable, the ability to distinguish between FH and RH was assessed by receiver operating characteristic (ROC) curve construction followed by calculation of the area under the curve (AUC). A *P* value < 0.05 was considered statistically significant. All data were analyzed using SPSS software (ver. 17.0, IBM Corp., Armonk, NY, USA).

## 3. Results

The study initially enrolled 351 patients with heartburn and normal upper gastrointestinal endoscopy who underwent HRIM and MII/pH between December 2014 and December 2016, of whom 111 had abnormal acid exposure and 2 had nutcracker esophagi and were thus excluded. The 238 patients with normal acid exposure, made up of 147 patients diagnosed with FH and 91 with RH by MII/pH, were evaluated. A total of 36 HVs enrolled during the same period were also evaluated. The three groups did not differ in terms of age or sex.

Table 1 compares the demographic data and HRIM results among the three groups. The FH and RH patients exhibited similar LESP, LESRP, IRP, UESP, DEA, DCI, TBTT, and CBTR values. Compared to the FH patients, the RH patients had significantly greater rates of IEM and fragmented peristalsis, more herniation, and a lower DCI value. Compared to HV and FH subjects, RH patients had significantly greater IEM and FP rates and lower DCI values.

Table 2 compares the MII/pH results among the three groups. The FH and RH patients had similar recumbent acid exposure and bolus exposure time. Compared to the FH patients, the RH patients had significantly greater DeMeester scores, total acid exposure, and total bolus exposure time; more proximal total and distal total reflux events; a lower PSPW index; and lower MNBI values. Compared to HV and FH subjects, the RH patients had significantly more proximal total reflux and distal total reflux events, a lower PSPW index, and lower MNBI values.

Table 3 shows the results of stepwise linear regression analyses. The number of distal total reflux events was a positive predictor of RH; the PSPW index and MNBI values were negative predictors of RH.

Table 4 shows the results of stepwise linear regression seeking predictors of IEM. Belch SAP positivity and the total bolus exposure were positive predictors of IEM; CBTR and UESP values were negative predictors.

**Figure 1:**
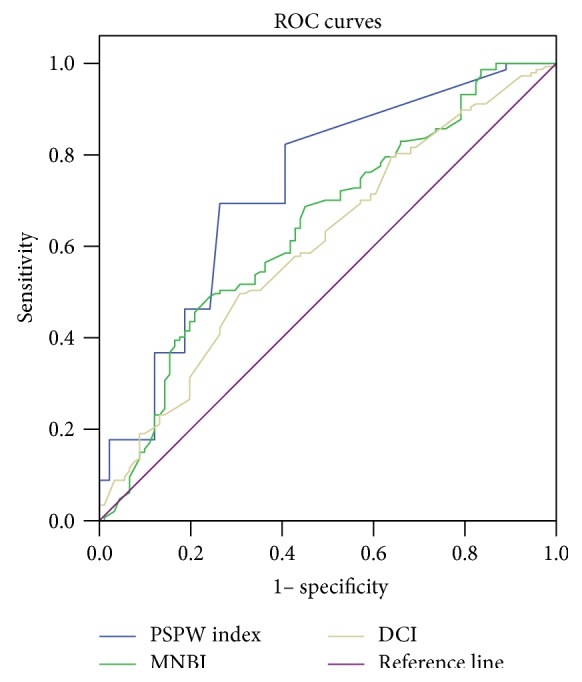
Receiver operating characteristic (ROC) curves for functional heartburn (FH) and reflux hypersensitivity (RH). On ROC analysis, the postreflux swallow-induced peristaltic wave index (the PSPW index), the mean nocturnal baseline impedance (MNBI), and the distal contractile integral (DCI) yielded areas under the curves of 0.728 (95% CI 0.661–0,796), 0.643 (95% CI 0.570–0.716), and 0.605 (95% CI 0.531–0.678), respectively, thus significantly distinguishing FH from RH.

**Figure 2:**
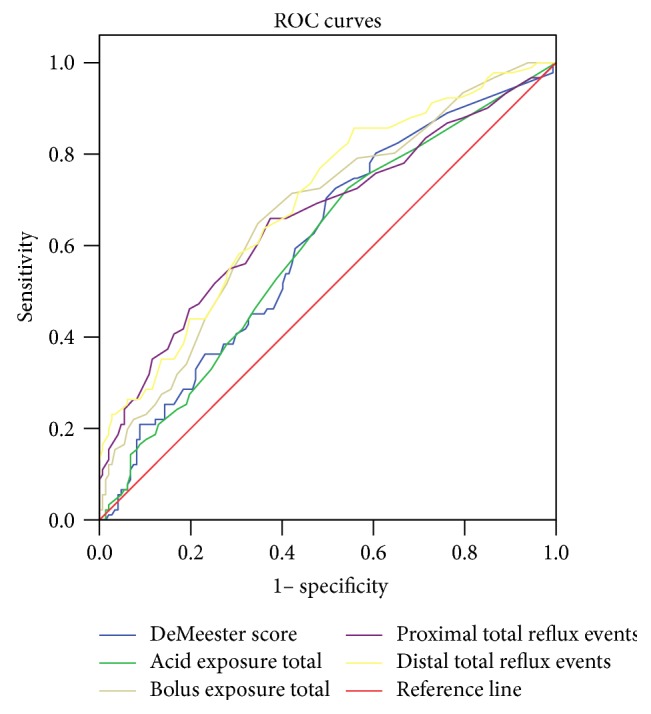
Receiver operating characteristic (ROC) curves for reflux hypersensitivity (RH) and functional heartburn (FH). On ROC analysis, the DeMeester score, total acid exposure, total bolus exposure, and the numbers of proximal total and distal total reflux events yielded areas under the curves of 0.607 (95% CI 0.534–0.680), 0.596 (95% CI 0.522–0.669), 0.671 (95% CI 0.601–0.741), 0.662 (95% CI 0.589–0.736), and 0.697 (95% CI 0.529–0.765), respectively, thus significantly distinguishing RH from FH.

In ROC analyses, the PSPW index, MNBI values, DCI, DeMeester score, total acid exposure, total bolus exposure, and the numbers of proximal total reflux and distal total reflux events yielded areas under the curve of 0.728 (95% CI 0.661–0,796), 0.643 (95% CI 0.570–0.716), 0.605 (95% CI 0.531–0.678), 0.607 (95% CI 0.534–0.680), 0.596 (95% CI 0.522–0.669), 0.671 (95% CI 0.601–0.741), 0.662 (95% CI 0.589–0.736), and 0.697 (95% CI 0.529–0.765), respectively, thus significantly distinguishing between FH and RH (Figures 1 and 2).

## 4. Discussion

The majority of patients complaining of heartburn have normal endoscopic examination results [16]. One recent study reported that 91% of patients with suspected GERD had normal endoscopic results, and approximately one-third of these patients were diagnosed with FH by pH testing [17]. As reflux events are not temporally associated with the generation of symptoms, the extent to which FH is a truly esophageal phenomenon, as opposed to a neuropathic or psychosomatic issue, is unclear. Although we differentiate RH from FH, some symptoms of FH may be related to visceral hypersensitivity of esophageal pain receptors to normal stimuli [2]. Abnormal responses to balloon distension and intraesophageal acid perfusion with abnormally high levels of pain have been observed in patients with FH [18]. Central neural mechanisms have also been postulated to play a role in FH [18, 19]. Patterns of cortical-evoked potentials in FH patients have been identified, which may be further evidence of central afferent sensitization. The importance of psychiatric factors in the generation of FH symptoms may also be considered. Patients with FH have high rates of anxiety, with fewer social support structures, compared to patients showing good correlation between reflux events and symptoms [20].

IEM, also known as esophageal hypocontractility, is a manometric pattern characterized by ineffective swallows with poor bolus transit in the distal esophagus. The Chicago Classification v3.0 defines IEM on Clouse plots using a DCI < 450 mmHg/s/cm, with greater than 50% ineffective swallows, and IEM is prevalent in GERD patients [21, 22]. We also found that FH patients had a greater rate of IEM than HVs. IEM is associated with the presence of abnormal acid reflux, as assessed by 24-hour esophageal pH recording, regardless of the presence of a defective LES, hiatus hernia, or esophagitis [23]. Stepwise linear regression showed that belch SAP positivity and total bolus exposure were positive predictors of IEM and CBTR and UESP values were negative predictors.

Defects in peristaltic wave integrity impair bolus transit and prolong esophageal acid exposure [24]. Bulsiewicz et al. [25] reported that longer breaks in the peristaltic wave predicted incomplete bolus clearance. Ribolsi et al. [26] found that weak peristalsis associated with a large break was associated with high-level acid exposure and delayed reflux clearance when GERD patients were placed in the supine position. The Chicago Classification version 3.0 defines fragmented peristalsis (FP) as a DCI > 450 mmHg/s/cm and a break >5 cm between the 20 mmHg isobaric contours, combined with ineffective swallows constituting >50% of all swallows. We found that RH patients had a greater FP rate than FH patients.

In contrast to a Japanese study that found no difference between FH and RH patients [27], in our study, MII/pH was used to show that RH patients had greater acid exposure and total bolus exposure time and more proximal and distal reflux events than FH patients.

Recently, impedance values for evaluating esophageal chemical clearance (the PSPW index) and mucosal integrity (MNBI) have been proposed [13, 14]. After a reflux episode, esophageal clearance is primarily achieved by secondary peristalsis, which removes around 90% of the reflux and is elicited by stretch receptors in the esophageal lining (volume clearance); however, a neutral esophageal pH is restored only after a voluntary swallow elicited by an esophagosalivary reflex mediated through the vagal afferents and delivery of salivary bicarbonate (chemical clearance) [28]. Impedance monitoring allows the assessment of chemical clearance independent of volume clearance: a decrease in impedance originating in the upper esophagus and attaining the lower esophagus signal peristaltic saliva transit; this is a PSPW event [13]. The MNBI is the mean of three values obtained during 10 min during the night, accurately reflecting a 6 h bedtime period. MNBI is minimally influenced by swallowing activity, rather reflecting reflux-induced impairment of mucosal integrity [14]. Analysis of impedance-pH data based on the PSPW index and the MNBI increases diagnostic accuracy in patients with reflux disease (compared to pH-only data) [29]. Moreover, erosive reflux disease is associated with a lower PSPW index and a lower MNBI value than NERD [30].

In the present study, RH patients had a significantly lower PSPW index, lower MNBI and DCI values, greater IEM and FP rates, a higher DeMeester score, longer acid exposure and bolus exposure times, and more proximal and distal reflux events than FH patients. Therefore, apart from acid suppression, different treatments may be appropriate for RH and FH patients.

As transient LES relaxation is the principal mechanism underlying all forms of reflux, directed therapy seeking to reduce the numbers of such events appears to be the next logical step when PPIs and H2RAs fail. However, despite aggressive pharmaceutical testing over the past 10 years, the only useful compound available is baclofen, a *γ*-aminobutyric acid type B (GABAB) agonist that has been used for many years to treat spastic muscle disorders. Baclofen reduces the numbers of postprandial acid and nonacid reflux events by inhibiting transient LES relaxation and reduces the symptoms of reflux [31]. In GERD patients, baclofen significantly increases the postprandial LES pressure and prevents an increase in the number of transient LES relaxations but does not act on “gastric acid pocket” extensions into the distal esophagus [32].

We have not prescribed baclofen, but we have experienced some success with combinations of PPI and mosapride or trimebutine. In an earlier study, we found that 18 healthy volunteers treated with mosapride for 7 days exhibited significant increases in DEA, DCI, and CBTR values and a significant decrease in TBTT. In addition, 16 GERD patients with IEM treated with a combination of PPI and trimebutine for 2 weeks exhibited significant increases in DEA, DCI, and LESP values. Compared to PPI treatment alone, GERD patients treated with a combination of PPI and mosapride reported that upper abdominal pain, belching, and total GERD symptom scores improved more rapidly. However, the endoscopic healing rates (scored using the Los Angeles classification) were similar between the two groups [33]. In another study, the combination treatment afforded less relief from reflux symptoms in NERD compared to erosive esophagitis patients [34]. Compared to omeprazole treatment alone, GERD patients with IBS given a combination of PPI and trimebutine for 3 months report significant improvements in both GERD and IBS symptoms and in the extent of erosive esophagitis [35].

Lifestyle changes and acid suppression are often recommended for FH patients, but the supporting data are limited. Unfortunately, no well-designed clinical trial has yet explored the pharmaceutical treatment options. Modulation of pain perception and alternative therapies may be potentially useful for FH patients. Because of the chronic nature of FH, it is essential to reassure patients that the clinical course is benign [2].

Our study has some limitations. First, all of the subjects were recruited from two centers in one city and no symptom severity score was assessed, which might result in potential selection bias. Furthermore, the small number of patients limited the statistical power of the study. However, we are the first to compare esophageal function tests between FH and RH in Chinese patients.

In summary, compared to HV and FH patients, RH patients had a greater IEM, a higher fragmented peristalsis rate, greater total bolus exposure, more proximal total and distal total refluxes, greater impairment of chemical clearance, and less mucosal integrity. ROC curves showed values of DCI, DeMeester score, acid exposure total, bolus exposure total, proximal and distal total reflux events, PSPW index, and MNBI that could identify between RH and FH. By using the above-described parameters, HRIM and MII/pH assays could be used to correctly classify RH and FH and hence allow physicians to provide adequate relief from associated symptoms.

## Figures and Tables

**Table 1:** Demographic data and high-resolution manometry and impedance results.

Items	FH	RH	Independent samples *t-*test, chi-square test, or Fisher's exact test	HV	ANOVA, chi-square test, or Fisher's exact test
	*n* = 147	*n* = 91		*n* = 36	
Age (mean ± SD, years)	57.5 ± 47.0	51.0 ± 12.8	*P* = 0.197	54.8 ± 12.3	*P* = 0.381
Male/female (*n*)	56/91	43/48	*P* = 0.164	16/20	*P* = 0.361
Dysphagia (*n*, %)	5 (3.4)	1 (1.0)	*P* = 0.258	0 (0)	*P* = 0.502
Belching (*n*, %)	12 (8.1)	25 (27.4)	*P* < 0.001	2 (5.5)	*P* < 0.001
Globus (*n*, %)	13 (8.8)	4 (4.3)	*P* = 0.150	1 (2.7)	*P* = 0.350
Chest pain (*n*, %)	24 (16.3)	10 (10.9)	*P* = 0.341	0 (0)	*P* = 0.011
Upper abdominal pain (*n*, %)	8 (5.4)	4 (4.3)	*P* = 0.511	3 (8.3)	*P* = 0.650
Abdominal distension (*n*, %)	5 (3.4)	1 (1.0)	*P* = 0.258	2 (5.5)	*P* = 0.285
LESP (mean ± SD, mmHg)	17.3 ± 10.3	17.1 ± 10.5	*P* = 0.878	22.1 ± 9.4	*P* = 0.941
LESL (mean ± SD, cm)	3.8 ± 0.9	3.7 ± 0.7	*P* = 0.209	3.9 ± 0.9	*P* = 0.337
LESRP (mean ± SD, mmHg)	5.2 ± 4.6	4.5 ± 4.2	*P* = 0.237	5.7 ± 4.3	*P* = 0.329
IRP (mean ± SD, mmHg)	8.1 ± 4.7	7.9 ± 5.2	*P* = 0.750	7.3 ± 4.5	*P* = 0.679
UESP (mean ± SD, mmHg)	82.8 ± 31.5	81.7 ± 35.9	*P* = 0.807	88.8 ± 36.3	*P* = 0.548
DEA (mean ± SD, mmHg)	68.3 ± 30.5	61.9 ± 26.2	*P* = 0.097	78.3 ± 36.4	*P* = 0.020
DCI (mean ± SD, mmHg·s·cm)	806.3 ± 752.5	575.7 ± 495.1	*P* = 0.010	1060.0 ± 635.5	*P* = 0.001
Ineffective esophageal motility (*n* (%))	40 (27.2)	38 (41.7)	*P* = 0.023	0 (0)	*P* < 0.001
Fragmented peristalsis (*n* (%))	5 (3.4)	9 (9.9)	*P* = 0.049	0 (0)	*P* = 0.037
Total bolus transit time, s	6.5 ± 1.4	6.5 ± 1.3	*P* = 0.805	6.2 ± 1.1	*P* = 0.491
Complete bolus transit rate, %	75.4 ± 30.9	72.7 ± 32.1	*P* = 0.521	94.7 ± 11.5	*P* = 0.001
Hiatus hernia (*n* (%))	4 (2.7)	8 (8.8)	*P* = 0.063	1 (2.8)	*P* = 0.108

HV: healthy volunteers; FH: functional heartburn; RH: reflux hypersensitivity; LESP: lower esophageal sphincter pressure; LESL: lower esophageal sphincter length; LESRP: lower esophageal sphincter residual pressure; IRP: integrated relaxation pressure; UESP: upper esophageal sphincter pressure; DEA: distal esophageal amplitude; DCI: distal contractile integral.

**Table 2:** Results of 24-hour multichannel intraluminal impedance and pH recording.

Items	FH	RH	Independent sample *t-*test, chi-square test, or Fisher's exact test	HV	ANOVA, chi-square test, or Fisher's exact test
	*n* = 147	*n* = 91		*n* = 36	
DeMeester	2.7 ± 2.4	3.3 ± 2.4	*P* = 0.043	3.0 ± 2.4	*P* = 0.124
Acid exposure upright (%)	1.0 ± 1.4	1.4 ± 1.4	*P* = 0.036	1.4 ± 1.7	*P* = 0.100
Acid exposure recumbent (%)	0.1 ± 0.2	0.1 ± 0.2	*P* = 0.445	0.1 ± 0.2	*P* = 0.617
Acid exposure total (%)	0.6 ± 0.7	0.8 ± 0.8	*P* = 0.044	0.7 ± 0.9	*P* = 0.130
Bolus exposure upright (%)	1.8 ± 1.5	2.6 ± 2.0	*P* = 0.001	1.9 ± 1.4	*P* = 0.002
Bolus exposure recumbent (%)	0.3 ± 0.7	0.4 ± 0.7	*P* = 0.200	0.2 ± 0.4	*P* = 0.268
Bolus exposure total (%)	0.9 ± 0.8	1.5 ± 1.1	*P* < 0.001	1.0 ± 0.7	*P* < 0.001
Proximal acid event (*n*)	5.6 ± 5.6	9.8 ± 8.9	*P* < 0.001	5.4 ± 4.4	*P* < 0.001
Proximal nonacid event (*n*)	6.9 ± 5.6	11.6 ± 10.8	*P* < 0.001	6.8 ± 5.7	*P* < 0.001
Proximal total reflux event (*n*)	12.3 ± 9.0	20.9 ± 16.8	*P* < 0.001	12.2 ± 7.5	*P* < 0.001
Distal acid reflux event (*n*)	8.4 ± 8.0	13.5 ± 11.2	*P* < 0.001	8.6 ± 7.5	*P* < 0.001
Distal nonacid reflux event (*n*)	16.6 ± 11.1	25.0 ± 15.3	*P* < 0.001	16.8 ± 9.8	*P* < 0.001
Distal total reflux event (*n*)	25.0 ± 14.4	38.5 ± 20.9	*P* < 0.001	25.1 ± 14.4	*P* < 0.001
PSPW index (%)	47.0 ± 17.9	33.8 ± 15.8	*P* < 0.001	73.6 ± 11.7	*P* < 0.001
MNBI (ohms)	2972.0 ± 775.6	2485.3 ± 939.2	*P* < 0.001	3290.1 ± 613.5	*P* < 0.001

HV: healthy volunteers; FH: functional heartburn; RH: reflux hypersensitivity; PSPW index: postreflux swallow-induced peristaltic wave index; MNBI: mean nocturnal baseline impedance.

**Table 3:** Results of stepwise linear regression analyses seeking predictors of reflux hypersensitivity (*n* = 238).

Items	Unstandardized coefficients		*P* value
	B	SE	
Constant	2.654	0.113	*P* < 0.001
Distal total reflux event	0.008	0002	*P* < 0.001
PSPW index	−0.007	0.002	*P* < 0.001
MNBI	−8.392E-5	0.000	*P* = 0.014

PSPW index: postreflux swallow-induced peristaltic wave index; MNBI: mean nocturnal baseline impedance.

**Table 4:** Results of stepwise linear regression analyses seeking predictors of ineffective esophageal motility (*n* = 238).

Items	Unstandardized coefficients		*P* value
	B	SE	
Constant	1.867	0.121	*P* < 0.001
Complete bolus transit rate	−0.092	0.007	*P* < 0.001
SAP-positive belch	0.182	0.066	*P* = 0.006
UESP	−0.002	0.001	*P* = 0.023
Bolus exposure total	0.049	0.023	*P* = 0.031

SAP: symptom association probability; UESP: upper esophageal sphincter pressure.
